# Identifying Predictive Risk Factors for Future Cognitive Impairment Among Chinese Older Adults: Longitudinal Prediction Study

**DOI:** 10.2196/53240

**Published:** 2024-03-22

**Authors:** Collin Sakal, Tingyou Li, Juan Li, Xinyue Li

**Affiliations:** 1 School of Data Science, City University of Hong Kong, Hong Kong, China; 2 Center on Aging Psychology, Key Laboratory of Mental Health, Institute of Psychology, Chinese Academy of Sciences, Beijing, China

**Keywords:** cognitive impairment, China, prediction, predictions, predict, predictor, predictors, risk, risks, population, demographic, demographics, gerontology, geriatric, geriatrics, older adult, older adults, elder, elderly, older person, older people, ageing, aging, MCI, cognitive, cognition, machine learning, variable, variables, model, models, mild cognitive impairment

## Abstract

**Background:**

The societal burden of cognitive impairment in China has prompted researchers to develop clinical prediction models aimed at making risk assessments that enable preventative interventions. However, it is unclear what types of risk factors best predict future cognitive impairment, if known risk factors make equally accurate predictions across different socioeconomic groups, and if existing prediction models are equally accurate across different subpopulations.

**Objective:**

This paper aimed to identify which domain of health information best predicts future cognitive impairment among Chinese older adults and to examine if discrepancies exist in predictive ability across different population subsets.

**Methods:**

Using data from the Chinese Longitudinal Healthy Longevity Survey, we quantified the ability of demographics, instrumental activities of daily living, activities of daily living, cognitive tests, social factors and hobbies, psychological factors, diet, exercise and sleep, chronic diseases, and 3 recently published logistic regression–based prediction models to predict 3-year risk of cognitive impairment in the general Chinese population and among male, female, rural-dwelling, urban-dwelling, educated, and not formally educated older adults. Predictive ability was quantified using the area under the receiver operating characteristic curve (AUC) and sensitivity-specificity curves through 20 repeats of 10-fold cross-validation.

**Results:**

A total of 4047 participants were included in the study, of which 337 (8.3%) developed cognitive impairment 3 years after baseline data collection. The risk factor groups with the best predictive ability in the general population were demographics (AUC 0.78, 95% CI 0.77-0.78), cognitive tests (AUC 0.72, 95% CI 0.72-0.73), and instrumental activities of daily living (AUC 0.71, 95% CI 0.70-0.71). Demographics, cognitive tests, instrumental activities of daily living, and all 3 recreated prediction models had significantly higher AUCs when making predictions among female older adults compared to male older adults and among older adults with no formal education compared to those with some education.

**Conclusions:**

This study suggests that demographics, cognitive tests, and instrumental activities of daily living are the most useful risk factors for predicting future cognitive impairment among Chinese older adults. However, the most predictive risk factors and existing models have lower predictive power among male, urban-dwelling, and educated older adults. More efforts are needed to ensure that equally accurate risk assessments can be conducted across different socioeconomic groups in China.

## Introduction

China’s aging population has led to cognitive impairment becoming increasingly burdensome to society [1,2]. In 2020, more than 68 million Chinese older adults had mild cognitive impairment, dementia, or Alzheimer disease [3]. The economic and social burden of cognitive impairment has led to calls for improving risk assessments and prioritizing early diagnoses [1,3]. Given China’s limited number of geriatric psychiatrists, researchers have turned to developing prediction models to identify older adults at risk of cognitive impairment for preventative interventions [4-8]. However, no study has compared the predictive ability of known risk factors side by side, and our understanding of which factors are the most useful for developing prediction models is limited. Furthermore, population characteristics vary widely across China, but it is unknown which risk factors are the most predictive in different socioeconomic groups, and existing prediction models have primarily been tested in the general population alone. To understand how to best predict future cognitive impairment and to develop more targeted prediction models for population subgroups, the predictive ability of known risk factors and existing prediction models must be quantified and compared across different subsets of the Chinese population.

A plethora of modifiable and nonmodifiable risk factors that are associated with cognitive impairment among Chinese older adults have been identified. Previous studies have found that increased age, limited functional independence, alcohol consumption, hypertension, and depression are significantly associated with cognitive impairment [3,9-12]. Protective factors have also been identified, namely, good sleep quality, sleeping sufficiently for many hours per night, exercise, and increased social participation [3,9,13-17]. It is also known that the prevalence of cognitive impairment in China differs across population subsets such as male and female individuals, rural and urban dwellers, and older adults with different levels of education [3]. Explanations for such discrepancies include different social patterns and literacy rates across the sexes and across regions with varying degrees of rurality, rates of depression in rural areas, and levels of education [3]. More years of education has also been associated with a greater cognitive reserve, which protects against future impairment [3]. Nevertheless, it is unclear which risk factors are the most useful for predicting future cognitive impairment across different subpopulations in China. Although we have a reasonable understanding of which characteristics make a person more likely to develop cognitive impairment, our understanding of which parts of a person’s health profile most accurately predict their risk of developing cognitive impairment is limited.

In addition to known risk factors, it is unclear if existing prediction models for future cognitive impairment are equally accurate across different socioeconomic groups in China. Several published models have reported areas under the receiver operating characteristic curve (AUCs) greater than 0.80 in their development cohorts [5,7,8], but each model has only been tested on the general population. Nearly all existing models make predictions by leveraging measures of cognition, age, and education. Additional covariates vary from model to model and include factors such as instrumental activities of daily living (IADL), hobbies such as gardening and watching television, marital status, and others. Examining the predictive ability of existing models across population subsets would allow us to identify where more efforts are needed to improve risk assessments for cognitive impairment, further our understanding of which subpopulations are more difficult to conduct risk assessments within, and provide a more thorough evaluation of existing prediction models than has been reported previously.

In this study, we quantified the ability of 9 risk factor groups and 3 existing models to predict future cognitive impairment among Chinese older adults. We examined how well demographics, IADL, activities of daily living (ADL), cognitive tests, social factors and hobbies, psychological factors, diet, exercise and sleep, chronic diseases, and 3 recently published models predict future cognitive impairment in the general population and among male, female, rural-dwelling, urban-dwelling, educated, and not formally educated older adults. To our knowledge, this study is the first to comprehensively compare the ability of known risk factors to predict future cognitive impairment and the first seeking to identify which subsets of the Chinese population need greater attention to improve the accuracy of risk assessments.

## Methods

### Data Source and Study Design

The Chinese Longitudinal Healthy Longevity Survey (CLHLS) is a prospective cohort study of Chinese older adults that contains information on demographics, cognitive function, lifestyle factors, chronic diseases, and more [18,19]. The CLHLS began in 1998, and follow-up surveys have been conducted every 2-3 years since. The data include older adults from 23 of China’s provinces that together make up 85% of the country’s total population.

We used the 2011 and 2014 CLHLS waves in our study. Baseline characteristics were gathered from the 2011 survey and used to predict if an individual became cognitively impaired by 2014. CLHLS participants younger than the age of 60 years or those with cognitive impairment at baseline were excluded. Sample size calculations were conducted following the methodology for multivariable prediction models by Riley et al [20]. This study is presented following the TRIPOD (Transparent Reporting of a Multivariable Prediction Model for Individual Prognosis or Diagnosis) guidelines where appropriate [21,22].

### Ethical Considerations

The CLHLS received ethics approval from the Duke University Institutional Review Board (Pro00062871) and Peking University’s Biomedical Ethics Committee (IRB00001052–13,074). Written informed consent was given by all participants prior to the survey interviews. This study secondarily analyzed anonymized data from the CLHLS.

### Measuring Cognitive Function

Cognition was assessed through the Chinese-language version of the Mini Mental State Examination (MMSE) [23]. MMSE scores range from 0 to 30, with lower scores indicating worse cognitive function. We adopted education-specific cutoffs that have been previously validated in the Chinese older adult population to indicate cognitive impairment [24]. Those with no formal education and MMSE scores less than 18 were labeled as cognitively impaired, as were those with 1-6 years of education with scores less than 21 and those with more than 6 years of education with scores less than 25 [24].

### Risk Factor Groups

#### Overview

A total of 9 groups containing known risk factors for cognitive impairment were considered in this study: demographics, ADL, IADL, cognitive tests, social factors and hobbies, psychological factors, exercise and sleep, diet, and chronic diseases. The risk factor groups were chosen by selecting parts of a person’s health profile previously found to be associated with developing cognitive impairment [9]. Each group is briefly described below, and a complete list of the variables in each group can be found in Multimedia Appendix 1.

#### Demographics Group

The demographics group contained each individual’s age, sex, years of education, household income, marital status, and residence location (city, town, or rural area).

#### ADL Group

The ADL group included each person’s ability to bathe, get dressed, use the toilet, get in and out of bed, control urination and bowel movements, and eat food.

#### IADL Group

The IADL group covered tasks that require thinking, organizational, and physical independence. The IADL group included an older adult’s ability to visit neighbors, go shopping, cook, wash clothes, walk continuously for 1 km, lift a bag of groceries, crouch and stand up, and take public transportation.

#### Cognitive Tests Group

The cognitive tests group included scores from each subsection of the MMSE: orientation, naming, registration, calculation, attention, recall, and language. Scores from each section were included as separate variables.

#### Social Factors and Hobbies Group

The social factors and hobbies group included whether a person grows vegetables, gardens, reads newspapers and books, looks after pets or animals, plays cards or mahjong, and participates in social activities.

#### Psychological Factors Group

The psychological factors group included the following factors that primarily relate to depression and anxiety: whether a person is generally optimistic, keeps their belongings organized, is generally anxious, is often lonely, makes decisions independently, feels useless with age, was happier when they were younger, and felt sad for more than 2 consecutive weeks over the past year.

#### Exercise and Sleep Group

The exercise and sleep group included whether someone currently exercises, whether they used to exercise, as well as the self-reported duration and quality of sleep.

#### Diet Group

The diet group contained information on each person’s staple food; if they eat fresh fruits and vegetables; the main flavor of the dishes they cook; how frequently they consume meat, fish, eggs, sugar, and tea; if they consume alcohol; the type of alcohol they consume; and the frequency of alcohol consumption.

#### Chronic Diseases Group

The chronic diseases group included the presence or absence of hypertension, diabetes, heart disease, blood disease, and cardiovascular disease.

### Recreating Existing Prediction Models

Prediction models were selected based on the following criteria: the model was developed for use in China, was reproducible using the CLHLS, and had an AUC of >0.75 during development. We selected 3 models published in Zhou et al [8], Hu et al [5], and Wang et al [7]. Each model was developed for use in the general Chinese population and showed excellent predictive performance (AUC>0.80) during development. All the models we recreated were based on logistic regression, which returns predictions by summing weighted values of each covariate before the sum is passed through the logistic function to produce predicted probabilities between 0 and 1. The logistic regression model recreated from Zhou et al [8] included age, a functional independence score based on ADL, baseline MMSE score, chewing ability, visual function, history of stroke, whether the participant watches TV or listens to the radio, and whether the participant grows flowers or raises pets. From Hu et al [5], the recreated model included age, marital status, IADL, and baseline MMSE score. Lastly, the model from Wang et al [7] included age; education; sex; ADL; baseline MMSE; and whether the participant gardens, reads newspapers or books, plays mahjong or cards, watches TV, or listens to the radio.

### Statistical Analysis

All analyses were performed using the R Statistical Software (version 4.0.5; R Foundation for Statistical Computing), and all code required to reproduce the analyses presented herein can be found on the web [25]. Predictive ability was quantified using AUC, sensitivity, and specificity. We assessed the predictive ability of each risk factor group using logistic regression models evaluated through 20 repeats of 10-fold cross validation, which has been recommended to obtain optimism-corrected performance metrics for prediction models [26]. This resulted in 200 training sets and 200 validation sets. All “I don’t know” or “refused” responses in the CLHLSs were set to missing, ordinal variables were integer encoded, and nonordinal categorical variables were dummy encoded. Missing values were imputed on each training and validation set separately using *k*-nearest neighbors imputation [27]. During each iteration of cross-validation, the data were split into training and validation sets before 9 logistic regression models, each containing all covariates in 1 particular risk factor group, were fit to the training data. Thereafter, each model was used to make predictions on the validation set for the general population and 6 subpopulations: male, female, rural-dwelling, urban-dwelling, educated, and not formally educated older adults. The same procedure was also followed for evaluating the prediction models from Zhou et al [8], Hu et al [5], and Wang et al [7]. Average AUCs and accompanying 95% CIs were calculated across the 200 validation-set AUCs for each model in this study. Sensitivity and specificity curves, 1 from each validation set, were also plotted for the risk factor group models.

## Results

Given a binary outcome, a population-level prevalence of 0.20, a conservatively estimated Cox-Snell *R*^2^ of 0.09, and 24 predictors in the largest risk factor group, the sample size required for this study was determined to be 1065 with 213 events to minimize the risk of overfitting, reduce the chance of overly optimistic performance metrics, and ensure that the models have sufficient data to estimate the overall risk of cognitive impairment in our sample. After excluding CLHLS participants with cognitive impairment at baseline and those younger than the age of 60 years, a cohort of 4047 Chinese older adults were included, of which 337 (8.3%) developed cognitive impairment. The average age of the cohort was 79.8 (SD 9.4) years, and 2037 (50%) were male. The group that developed cognitive impairment was older at baseline (89.1 vs 79.0 years) with a lower average baseline MMSE score (25.1 vs 27.7). A full description of the cohort’s characteristics can be found in Table 1, and the distribution of variables in each risk factor group can be found in Multimedia Appendix 2.

**Table 1.** Baseline cohort characteristics.

Characteristics		All participants (N=4047)	Developed cognitive impairment	
			Yes (n=337)	No (n=3710)
Age (y), mean (SD)		79.8 (9.4)	89.1 (9.8)	79.0 (8.9)
**Sex, n (%)**				
	Male	2037 (50.3)	130 (38.6)	1907 (51.4)
	Female	2010 (49.7)	207 (61.4)	1803 (48.6)
Years of schooling, mean (SD)		2.8 (3.7)	1.8 (3.2)	2.9 (3.7)
Household income (CN ¥; CN ¥1=US $0.14), mean (SD)		24,483.8 (25,778.6)	22,942.1 (23,198.5)	24,623.1 (25,997.7)
**Marital status, n (%)**				
	Married and living with spouse	2033 (50.3)	83 (24.6)	1950 (52.7)
	Married but not living with spouse	89 (2.2)	4 (1.2)	85 (2.3)
	Divorced	8 (0.2)	0 (0)	8 (0.2)
	Widowed	1862 (46.1)	246 (73)	1616 (43.7)
	Never married	46 (1.1)	4 (1.2)	42 (1.1)
**Residential status, n (%)**				
	City	665 (16.4)	57 (16.9)	608 (16.4)
	Town	1241 (30.7)	89 (26.4)	1152 (31.1)
	Rural area	2141 (52.9)	191 (56.7)	1950 (52.6)
Baseline MMSE^a^ score, mean S(D)		27.5 (2.8)	25.1 (3.6)	27.7 (2.6)
Follow-up MMSE score, mean (SD)		26.2 (5.2)	12.8 (6.1)	27.5 (2.8)

a MMSE: Mini Mental State Examination.

**Figure 1.**
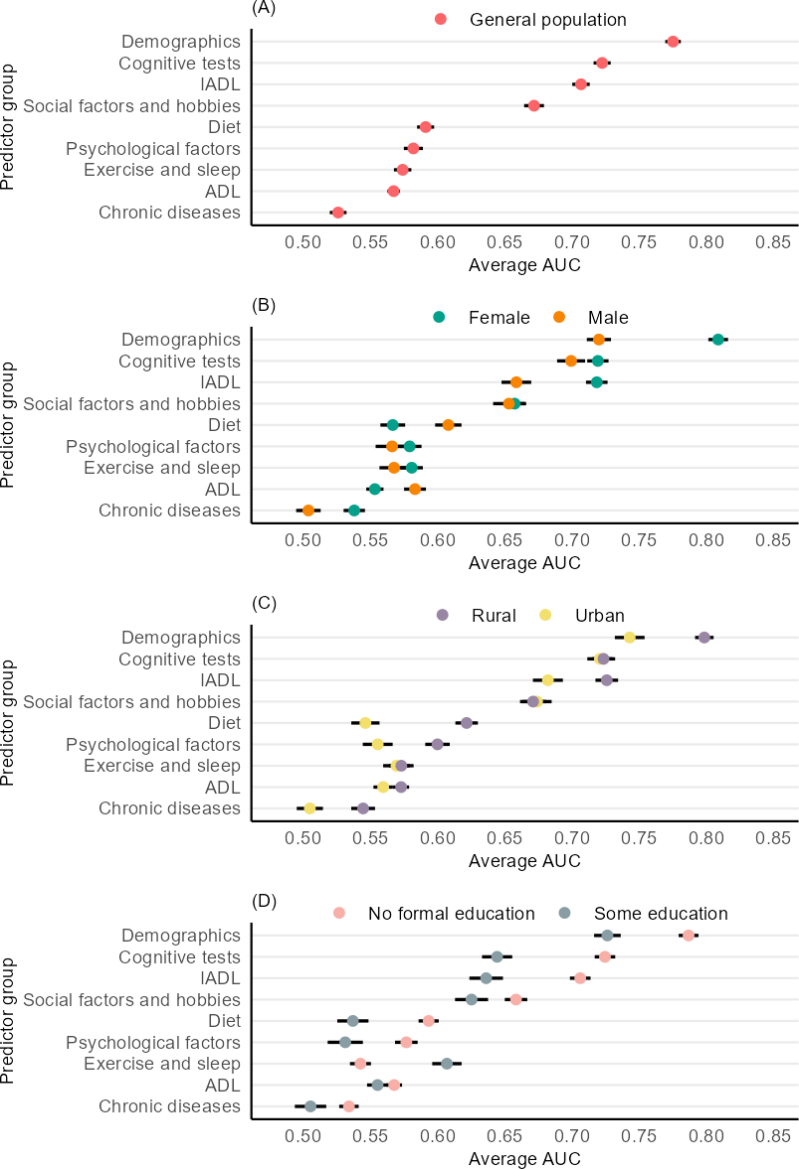
Average AUC by predictor group and target population. ADL: activities of daily living; AUC: area under the receiver operating characteristic curve; IADL: instrumental activities of daily living.

**Figure 2.**
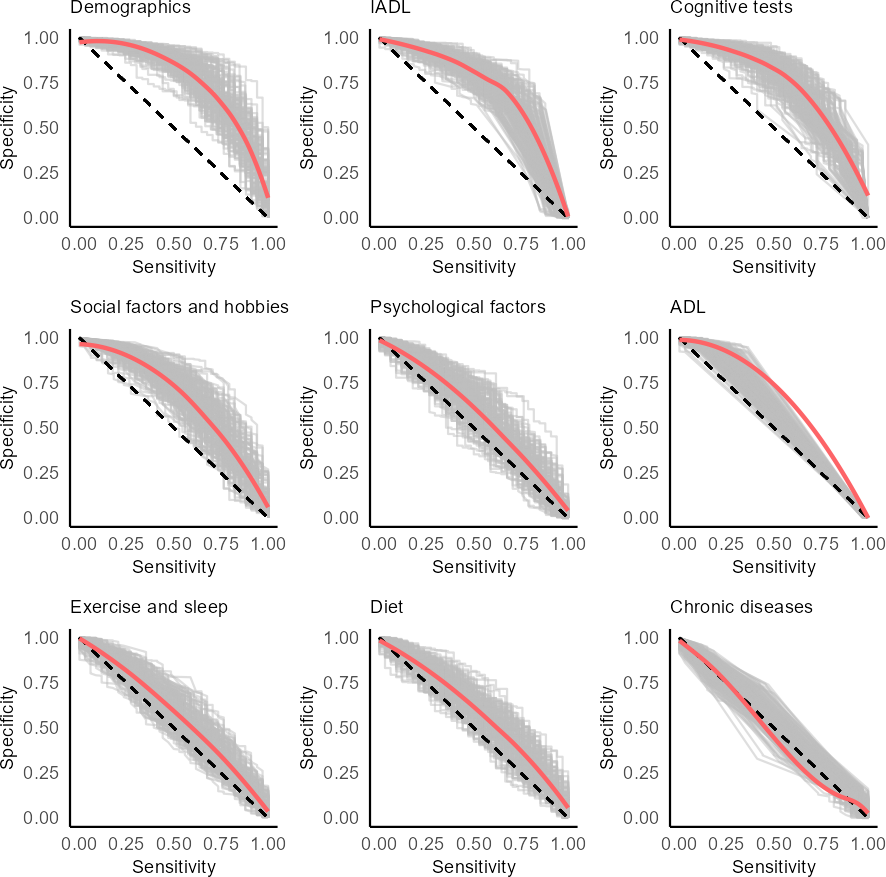
Sensitivity and specificity curves for predictions made in the general population. ADL: activities of daily living; IADL: instrumental activities of daily living.

As shown in Figure 1A and Table 2, demographics had the best predictive ability in the general population (AUC 0.78, 95% CI 0.77-0.78), followed by cognitive tests (AUC 0.72, 95% CI 0.72-0.73) and IADL (AUC 0.71, 95% CI 0.70-0.71). Social factors and hobbies had a moderate predictive ability (AUC 0.67, 95% CI 0.66-0.68), whereas diet, psychological factors, exercise and sleep, ADL, and chronic diseases all had average AUCs less than 0.60. Demographics, cognitive tests, and IADL also had the best sensitivity and specificity tradeoffs, as shown in Figure 2. By contrast, the sensitivity and specificity curves for the chronic diseases group showed that such risk factors only sometimes resulted in better-than-random predictions.

**Table 2.** Predictive ability by target population.

Model	Target population, AUC^a^ (95% CI)						
	General population	Male	Female	Rural	Urban	No formal education	Some education
Demographics	0.78 (0.77-0.78)	0.72 (0.71-0.73)	0.81 (0.80-0.82)	0.80 (0.79-0.81)	0.74 (0.73-0.75)	0.79 (0.78-0.79)	0.73 (0.72-0.74)
Cognitive tests	0.72 (0.72-0.73)	0.70 (0.69-0.71)	0.72 (0.71-0.73)	0.72 (0.71-0.73)	0.72 (0.71-0.73)	0.72 (0.72-0.73)	0.64 (0.63-0.66)
IADL^b^	0.71 (0.70-0.71)	0.66 (0.65-0.67)	0.72 (0.71-0.73)	0.73 (0.72-0.73)	0.68 (0.67-0.69)	0.71 (0.70-0.71)	0.64 (0.62-0.65)
Social factors and hobbies	0.67 (0.66-0.68)	0.65 (0.64-0.66)	0.66 (0.65-0.67)	0.67 (0.66-0.68)	0.67 (0.66-0.68)	0.66 (0.65-0.67)	0.63 (0.61-0.64)
Diet	0.59 (0.58-0.60)	0.61 (0.60-0.62)	0.57 (0.56-0.58)	0.62 (0.61-0.63)	0.55 (0.54-0.56)	0.59 (0.59-0.60)	0.54 (0.53-0.55)
Psychological factors	0.58 (0.57-0.59)	0.57 (0.55-0.58)	0.58 (0.57-0.59)	0.60 (0.59-0.61)	0.56 (0.54-0.57)	0.58 (0.57-0.59)	0.53 (0.52-0.54)
Exercise and sleep	0.57 (0.57-0.58)	0.57 (0.56-0.58)	0.58 (0.57-0.59)	0.57 (0.56-0.58)	0.57 (0.56-0.58)	0.54 (0.53-0.55)	0.61 (0.60-0.62)
ADL^c^	0.57 (0.56-0.57)	0.58 (0.58-0.59)	0.55 (0.55-0.56)	0.57 (0.57-0.58)	0.56 (0.55-0.57)	0.57 (0.56-0.57)	0.56 (0.55-0.56)
Chronic diseases	0.53 (0.52-0.53)	0.50 (0.49-0.51)	0.54 (0.53-0.55)	0.54 (0.54-0.55)	0.50 (0.50-0.51)	0.53 (0.53-0.54)	0.51 (0.49-0.52)
Wang et al [7]	0.80 (0.80-0.81)	0.78 (0.77-0.78)	0.82 (0.81-0.82)	0.82 (0.81-0.83)	0.78 (0.77-0.79)	0.81 (0.81-0.82)	0.75 (0.74-0.76)
Zhou et al [12]	0.80 (0.80-0.81)	0.78 (0.77-0.79)	0.82 (0.81-0.82)	0.82 (0.81-0.83)	0.78 (0.77-0.79)	0.82 (0.81-0.83)	0.75 (0.74-0.76)
Hu et al [5]	0.80 (0.80-0.81)	0.77 (0.77-0.78)	0.82 (0.81-0.83)	0.82 (0.81-0.83)	0.78 (0.77-0.79)	0.82 (0.81-0.82)	0.75 (0.74-0.76)

a AUC: area under the receiver operating characteristic curve.

b IADL: instrumental activities of daily living.

c ADL: activities of daily living.

Figure 1D shows that most risk factor groups had significantly higher AUCs when making predictions among older adults with no formal education compared to those with some education. The only exceptions were the ADL and exercise and sleep groups. Among those with no formal education, demographics, cognitive tests, and IADL had AUCs of 0.79 (95% CI 0.78-0.79), 0.72 (95% CI 0.72-0.73), and 0.71 (95% CI 0.70-0.71), respectively. When making predictions among those with some education, demographics, cognitive tests, and IADL had average AUCs of 0.73 (95% CI 0.72-0.74), 0.64 (95% CI 0.63-0.66), and 0.64 (95% CI 0.62-0.65), respectively.

**Figure 3.**
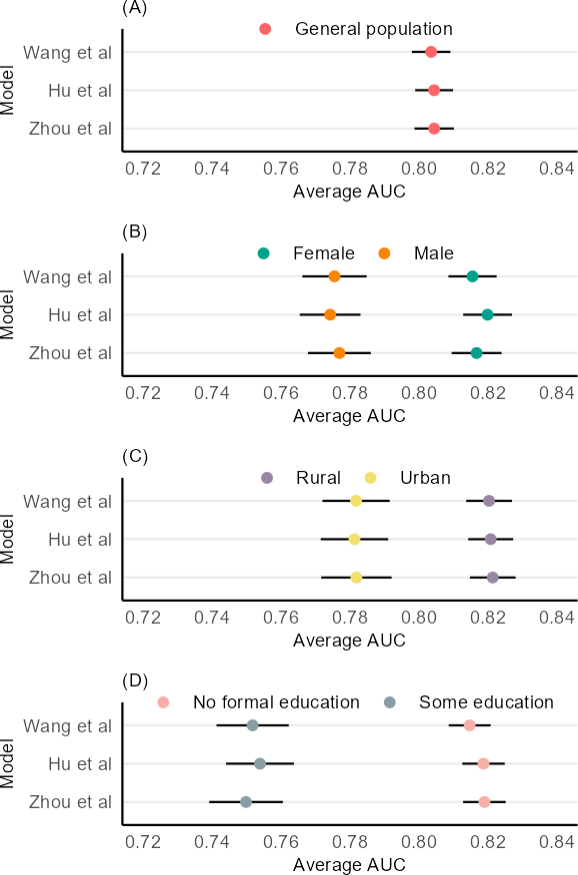
Predictive ability of existing models [5,7,8]. AUC: area under the receiver operating characteristic curve.

The existing prediction models recreated in this study all had good predictive ability in the general population. Each model had an average AUC of 0.80 (95% CI 0.80-80.1). However, each model had significantly higher AUCs when making predictions in female individuals compared to male individuals, in rural dwellers compared to urban dwellers, and in those with no formal education compared to those with some education. Complete results can be found in Table 2 and Figure 3. The TRIPOD checklist for this study can be found in Checklist 1.

Demographics had a significantly better predictive ability when making predictions among rural dwellers (AUC 0.80, 95% CI 0.79-0.81) compared to urban dwellers (AUC 0.74, 95% CI 0.73-0.75). Similarly, IADL showed a higher average AUC among rural dwellers (AUC 0.73, 95% CI 0.72-0.73) compared to urban dwellers (AUC 0.68, 95% CI 0.67-0.69). As shown in Figure 1C and Table 2, significantly higher AUCs among rural dwellers were also observed for the diet, psychological factors, and chronic diseases groups.

Demographics, cognitive tests, and IADL also had the highest average AUCs when making predictions for male and female individuals, although the predictive ability varied between the 2 sexes. The demographics group had a higher average AUC when making predictions in female individuals compared to male individuals (0.81, 95% CI 0.80-0.82 vs 0.72, 95% CI 0.71-0.73), as did the IADL group (0.72, 95% CI 0.71-0.73 vs 0.66, 95% CI 0.65-0.67) and the cognitive tests group (0.72, 95% CI 0.71-0.73 vs 0.70, 95% CI 0.69-0.71). The dietary group had a significantly higher AUC when making predictions among male individuals (0.61, 95% CI 0.60-0.62) compared to female individuals (0.57, 95% CI 0.56-0.58). No significant differences were observed for the social factors and hobbies group, and all other remaining groups has AUCs less than 0.60 for both male and female individuals. Full results can be found in Figure 1B and Table 2.

## Discussion

### Principal Findings

In this study, we quantified the ability of 9 risk factor groups and 3 prediction models to predict future cognitive impairment in the general Chinese population and 6 population subsets: male, female, rural-dwelling, urban-dwelling, educated, and not formally educated older adults. In the general population, the risk factor groups with the best predictive ability were demographics (AUC 0.78, 95% CI 0.77-0.78), cognitive tests (AUC 0.72, 95% CI 0.72-0.73), and IADL (AUC 0.71, 95% CI 0.70-0.71). The most predictive risk factors and the existing models performed inconsistently across socioeconomic groups and had significantly higher AUCs when making predictions for female individuals and older adults with no formal education compared to male individuals and older adults with some education.

Our study showed that the 3 existing prediction models had significantly lower AUCs when predicting future cognitive impairment among male, urban-dwelling, and educated Chinese older adults compared to female, rural-dwelling, and not formally educated older adults. Despite the only shared risk factors in all 3 models being age and baseline MMSE score, significant differences in predictive ability were consistent across every model. One explanation is that risk factor differences between those who developed cognitive impairment and those who did not were larger among the groups where more accurate predictions were made. For example, the difference in average age between female older adults who did and did not become cognitively impaired was 11.8 years, whereas for male older adults, it was 7.0 years. Similarly, among those with no formal education, the difference in baseline MMSE score between those with and without cognitive impairment at follow-up was 2.67 compared to 1.45 among those with some education. In addition, the prevalence of cognitive impairment in our sample was higher among female, rural-dwelling, and not formally educated older adults, meaning that the models had more events to learn from. Indeed, previous studies using nationally representative data have also reported higher prevalence estimates among these groups [3]. Our results indicate that targeted prediction models for specific socioeconomic groups are needed in China to make equally accurate risk assessments across sex, residential status, and education level. Several studies have called for such models [28,29], but as of this writing, none have been developed in China.

Out of the 9 risk factor groups, we found that demographics, cognitive tests, and IADL best predict future cognitive impairment in the general Chinese population and across sex, residential status, and education level. Demographics are often included in prediction models for cognitive impairment [28,30-32], and we suggest that they continue to be leveraged because of their predictive power and ease to collect. Associations between chronic diseases, ADL, psychological factors, and diet with cognitive impairment among Chinese older adults have been established [13,33-39], but such factors showed moderate predictive ability in our study. To our knowledge, dietary factors have not been incorporated into existing prediction models in China, but they had higher AUCs than commonly used risk factors such as psychological factors, ADL, and chronic diseases. In fact, chronic diseases did not make significantly better than random predictions among male (AUC 0.50, 95% CI 0.49-0.51), urban-dwelling (AUC 0.50, 95% CI 0.50-0.51), and not formally educated (AUC 0.51, 95% CI 0.49-0.52) older adults. Hence, in addition to providing a ranking of the most predictive risk factor groups, our study is the first to show that dietary factors warrant consideration when predicting future cognitive impairment among Chinese older adults.

Many risk factor groups had significantly different AUCs across population subsets. Similar to the existing models we recreated, our study revealed that the most predictive risk factors (demographics, cognitive tests, and IADL) had significantly higher AUCs when making predictions among female and not formally educated older adults compared to male and educated older adults. As was the case with the recreated models, this likely resulted from the distributions of risk factors being more separable between those who developed cognitive impairment and those who did not in the groups where more accurate predictions were made. Given the lack of available evidence, it is unclear whether the discrepancies in predictive ability found in our study generalize outside of China, and future work may seek to perform similar analyses elsewhere.

### Limitations

Our study has several limitations. The source code was not available for the models we selected to recreate in this study, but we explicitly followed all preprocessing, variable derivation, and model creation procedures described in the original papers during the model replication process. The AUCs of each model in the general population in this study were consistent with the reported AUCs in the original papers, suggesting that the models were properly recreated from scratch. To facilitate future research, we have further made our code publicly available. Second, the CLHLS is not nationally representative, although it does include older adults from 23 of China’s provinces. The exercise and sleep group did not include objective measurements of physical activity and sleep. Self-reported exercise and sleep are often inaccurate, and we suggest that the results be interpreted with caution for the exercise and sleep group. Lastly, the data used in this study were from 2011 to 2014. Future studies may wish to collect new data and further validate the results presented herein.

### Conclusions

Out of the 9 risk factor groups, our study found that demographics, cognitive tests, and IADL best predicted future cognitive impairment among Chinese older adults and had significantly better predictive ability among female and not formally educated older adults compared to male and educated older adults. Similarly, every existing model we recreated made significantly better predictions among female, rural-dwelling, and not formally educated older adults. Our study suggests that more targeted predictions models for cognitive impairment are needed to make equally accurate risk assessments across different socioeconomic groups in China and provides foundational evidence that can support variable selection for such models.

## Supplementary material

10.2196/53240 Multimedia Appendix 1 Predictors in each risk factor group and prediction model.

10.2196/53240 Multimedia Appendix 2 Cohort characteristics for each covariate from every risk factor group.

10.2196/53240 Checklist 1 TRIPOD (Transparent Reporting of a Multivariable Prediction Model for Individual Prognosis or Diagnosis) checklist.

## Abbreviations

ADL: activities of daily living
AUC: area under the receiver operating characteristic curve
CLHLS: Chinese Longitudinal Healthy Longevity Survey
IADL: instrumental activities of daily living
MMSE: Mini Mental State Examination
TRIPOD: Transparent Reporting of a Multivariable Prediction Model for Individual Prognosis or Diagnosis
