# Supplementary material for: Identifying Predictive Risk Factors for Future Cognitive Impairment Among Chinese Older Adults: Longitudinal Prediction Study
Source: JMIR Aging. 2024 Mar 22;7:e53240. doi: 10.2196/53240 (PMC11004610; doi:10.2196/53240)
Supplement: Multimedia Appendix 1 [file aging-v7-e53240-s001.docx]

**Multimedia Appendix 1. Predictors in each risk factor group and prediction model.**

**Table S1.** Predictors in each risk factor group

| **Risk factor group** | **Predictors** |
| --- | --- |
| Demographics | Age, sex, residential status, years of schooling, household income, marital status |
| IADLS | Ability to visit neighbors, go shopping, cook, wash clothes, walk continuously for 1km, lift a bag of groceries, crouch and stand up, take public transportation |
| Cognition | Scores from subsection of the MMSE: orientation, naming foods, immediate recall, delayed recall, calculation, drawing, language |
| Social Factors and Hobbies | If/how often a person grows vegetables, gardens, reads newspapers and books, looks after pets or animals, plays cards or mahjong, and participates in social activities |
| ADLs | Whether or not a person can get dressed, use the toilet, get in and out of bed, control urination and bowel movements, bathe, and eat independently |
| Psychological Factors | Whether a person is generally optimistic, organized, anxious, lonely, makes decisions independently, feels more useless as they age, was happier when they were younger, and felt sad for 2+ weeks consecutively over the last year |
| Exercise and Sleep | Current exercise status, if a person used to exercise, sleep duration, sleep quality |
| Diet | Staple food, fruit and vegetable consumption, main flavor of their dishes, frequency they consume meat, eggs, sugar and tea, alcohol consumption, type of alcohol consumed, and frequency of alcohol consumption |
| Chronic Diseases | If a person has hypertension, diabetes, heart disease, blood disease, or cardiovascular disease |

**Table S2**. Predictors included in each re-created model

| Model | Predictors |
| --- | --- |
| Wang (2022) | Age, education level, sex, ADLs, gardening participation, reads newspapers or books, plays mahjong/cards, watches TV or listens to the radio, baseline MMSE |
| Zhou (2021) | Age, ADL/IADL score, baseline MMSE, chewing ability, visual function, history of stroke, watches tv or listens to the radio, grows flowers or raises pets, baseline MMSE |
| Hu (2020) | Age, marital status, IADLs, baseline MMSE |
