# Supplementary material for: Identifying Predictive Risk Factors for Future Cognitive Impairment Among Chinese Older Adults: Longitudinal Prediction Study
Source: JMIR Aging. 2024 Mar 22;7:e53240. doi: 10.2196/53240 (PMC11004610; doi:10.2196/53240)
Supplement: Multimedia Appendix 2 [file aging-v7-e53240-s002.docx]

**Multimedia Appendix 2. Cohort characteristics for each covariate from every risk factor group.**

**Table S1.** Cohort characteristics – demographic factors

|  | No. (%) | | | |
| --- | --- | --- | --- | --- |
| **Characteristic** |  | **Developed Cognitive Impairments?** | | **Missing** |
|  | Everyone (n = 4047) | Yes (n = 337) | No (n = 3710) |  |
| Age, mean (SD), year | 79.8 (9.4) | 89.1 (9.8) | 79 (8.9) | 0 (0.0) |
| Residential Status |  |  |  | 0 (0.0) |
| City | 665 (16.4) | 57 (16.9) | 608 (16.4) |  |
| Town | 1241 (30.7) | 89 (26.4) | 1152 (31.1) |  |
| Rural | 2141 (52.9) | 191 (56.7) | 1950 (52.6) |  |
| Sex |  |  |  | 0 (0.0) |
| Male | 2037 (50.3) | 130 (38.6) | 1907 (51.4) |  |
| Female | 2010 (49.7) | 207 (61.4) | 1803 (48.6) |  |
| Years of schooling, mean (SD) | 2.8 (3.7) | 1.8 (3.2) | 2.9 (3.7) | 0 (0.0) |
| Total income of your household last year, mean (SD), CNY | 24483.8 (25778.6) | 22942.1 (23198.5) | 24623.1(25997.7) | 330 (8.2) |
| Current Marital Status |  |  |  | 9 (0.2) |
| Married and living with spouse | 2033 (50.3) | 83 (24.6) | 1950 (52.7) |  |
| Married but not living with spouse | 89 (2.2) | 4 (1.2) | 85 (2.3) |  |
| Divorced | 8 (0.2) | 0 (0) | 8 (0.2) |  |
| Widowed | 1862 (46.1) | 246 (73) | 1616 (43.7) |  |
| Never married | 46 (1.1) | 4 (1.2) | 42 (1.1) |  |

**Table S2.** Cohort characteristics – cognitive factors

|  | No. (%) | | | |
| --- | --- | --- | --- | --- |
| **Characteristic** |  | **Developed Cognitive Impairments?** | | **Missing** |
|  | Everyone (n = 4047) | Yes (n = 337) | No (n = 3710) |  |
| MMSE: orientation, mean (SD) | 4.9 (0.4) | 4.7 (0.7) | 4.9 (0.4) | 0 (0.0) |
| MMSE: naming foods, mean (SD) | 6.5 (1.1) | 6.1 (1.3) | 6.6 (1) | 0 (0.0) |
| MMSE: immediate recall, mean (SD) | 2.8 (0.5) | 2.7 (0.7) | 2.9 (0.5) | 0 (0.0) |
| MMSE: delayed recall, mean (SD) | 2.5 (0.9) | 2.2 (1.2) | 2.5 (0.9) | 0 (0.0) |
| MMSE: calculation, mean (SD) | 4.5 (1.3) | 3.6 (1.8) | 4.5 (1.2) | 0 (0.0) |
| MMSE: drawing, mean (SD) | 0.4 (0.5) | 0.2 (0.4) | 0.5 (0.5) | 0 (0.0) |
| MMSE: language, mean (SD) | 5.9 (0.5) | 5.6 (0.9) | 5.9 (0.5) | 0 (0.0) |

**Table S3.** Cohort characteristics – instrumental activities of daily living factors

|  | No. (%) | | | |
| --- | --- | --- | --- | --- |
| **Characteristic** |  | **Developed Cognitive Impairments?** | | **Missing** |
|  | Everyone (n = 4047) | Yes (n = 337) | No (n = 3710) |  |
| Ability to visit neighbors |  |  |  |  |
| Yes | 3817 (94.4) | 277 (82.2) | 3540 (95.5) | 2 (0.0) |
| A little difficult | 110 (2.7) | 21 (6.2) | 89 (2.4) |  |
| Unable to do so | 118 (2.9) | 39 (11.6) | 79 (2.1) |  |
| Ability to go shopping |  |  |  |  |
| Yes | 3557 (87.9) | 226 (67.1) | 3331 (89.8) | 1 (0.0) |
| A little difficult | 236 (5.8) | 42 (12.5) | 194 (5.2) |  |
| Unable to do so | 253 (6.3) | 69 (20.5) | 184 (5) |  |
| Ability to cook |  |  |  |  |
| Yes | 3568 (88.2) | 216 (64.1) | 3352 (90.4) | 2 (0.0) |
| A little difficult | 203 (5) | 45 (13.4) | 158 (4.3) |  |
| Unable to do so | 274 (6.8) | 76 (22.6) | 198 (5.3) |  |
| Ability to wash clothes |  |  |  |  |
| Yes | 3587 (88.7) | 236 (70) | 3351 (90.4) | 4 (0.1) |
| A little difficult | 205 (5.1) | 42 (12.5) | 163 (4.4) |  |
| Unable to do so | 251 (6.2) | 59 (17.5) | 192 (5.2) |  |
| Ability to walk continuously for 1km |  |  |  |  |
| Yes | 3102 (76.7) | 178 (52.8) | 2924 (78.8) | 1 (0.0) |
| A little difficult | 519 (12.8) | 70 (20.8) | 449 (12.1) |  |
| Unable to do so | 425 (10.5) | 89 (26.4) | 336 (9.1) |  |
| Ability to carry 5kg weight |  |  |  |  |
| Yes | 3137 (77.6) | 184 (54.6) | 2953 (79.6) | 2 (0.0) |
| A little difficult | 503 (12.4) | 70 (20.8) | 433 (11.7) |  |
| Unable to do so | 405 (10) | 83 (24.6) | 322 (8.7) |  |
| Ability to crouch and stand three times |  |  |  |  |
| Yes | 2877 (71.1) | 151 (44.8) | 2726 (73.5) | 3 (0.1) |
| A little difficult | 739 (18.3) | 104 (30.9) | 635 (17.1) |  |
| Unable to do so | 428 (10.6) | 82 (24.3) | 346 (9.3) |  |
| Ability to take public transportation |  |  |  |  |
| Yes | 2925 (72.4) | 139 (41.4) | 2786 (75.2) | 5 (0.1) |
| A little difficult | 523 (12.9) | 70 (20.8) | 453 (12.2) |  |
| Unable to do so | 594 (14.7) | 127 (37.8) | 467 (12.6) |  |

**Table S4.** Cohort characteristics – social factors

|  | No. (%) | | | |
| --- | --- | --- | --- | --- |
| **Characteristic** |  | **Developed Cognitive Impairments?** | | **Missing** |
|  | Everyone (n = 4047) | Yes (n = 337) | No (n = 3710) |  |
| Whether growing vegetables & do other field work at present |  |  |  |  |
| Almost everyday | 2242 (55.4) | 143 (42.4) | 2099 (56.6) | 0 (0.0) |
| Not daily, but once for a week | 455 (11.2) | 50 (14.8) | 405 (10.9) |  |
| Not weekly, but at least once for a month | 104 (2.6) | 11 (3.3) | 93 (2.5) |  |
| Not monthly, but sometimes | 205 (5.1) | 19 (5.6) | 186 (5) |  |
| Never | 1041 (25.7) | 114 (33.8) | 927 (25) |  |
| Whether doing garden work |  |  |  |  |
| Almost everyday | 814 (20.1) | 40 (11.9) | 774 (20.9) | 2 (0.0) |
| Not daily, but once for a week | 131 (3.2) | 4 (1.2) | 127 (3.4) |  |
| Not weekly, but at least once for a month | 65 (1.6) | 4 (1.2) | 61 (1.6) |  |
| Not monthly, but sometimes | 106 (2.6) | 10 (3) | 96 (2.6) |  |
| Never | 2929 (72.4) | 279 (82.8) | 2650 (71.5) |  |
| Whether reading newspapers/books at present |  |  |  |  |
| Almost everyday | 632 (15.6) | 31 (9.2) | 601 (16.2) | 3 (0.1) |
| Not daily, but once for a week | 222 (5.5) | 10 (3) | 212 (5.7) |  |
| Not weekly, but at least once for a month | 82 (2) | 3 (0.9) | 79 (2.1) |  |
| Not monthly, but sometimes | 191 (4.7) | 11 (3.3) | 180 (4.9) |  |
| Never | 2917 (72.1) | 282 (83.7) | 2635 (71.1) |  |
| Whether raising domestic animals/pets at present |  |  |  |  |
| Almost everyday | 1133 (28) | 64 (19) | 1069 (28.8) | 3 (0.1) |
| Not daily, but once for a week | 101 (2.5) | 6 (1.8) | 95 (2.6) |  |
| Not weekly, but at least once for a month | 66 (1.6) | 6 (1.8) | 60 (1.6) |  |
| Not monthly, but sometimes | 93 (2.3) | 10 (3) | 83 (2.2) |  |
| Never | 2651 (65.6) | 251 (74.5) | 2400 (64.7) |  |
| Whether playing cards/mah-jongg at present |  |  |  |  |
| Almost everyday | 383 (9.5) | 16 (4.7) | 367 (9.9) | 4 (0.1) |
| Not daily, but once for a week | 259 (6.4) | 12 (3.6) | 247 (6.7) |  |
| Not weekly, but at least once for a month | 91 (2.3) | 7 (2.1) | 84 (2.3) |  |
| Not monthly, but sometimes | 185 (4.6) | 6 (1.8) | 179 (4.8) |  |
| Never | 3125 (77.3) | 296 (87.8) | 2829 (76.3) |  |

|  | No. (%) | | | |
| --- | --- | --- | --- | --- |
| **Characteristic** |  | **Developed Cognitive Impairments?** | | **Missing** |
|  | Everyone (n = 4047) | Yes (n = 337) | No (n = 3710) |  |
| Frequency of watching tv or listen to radio at present |  |  |  |  |
| Almost everyday | 2779 (68.7) | 150 (44.5) | 2629 (70.9) | 2 (0.0) |
| Not daily, but once for a week | 387 (9.6) | 41 (12.2) | 346 (9.3) |  |
| Not weekly, but at least once for a month | 116 (2.9) | 14 (4.2) | 102 (2.8) |  |
| Not monthly, but sometimes | 147 (3.6) | 18 (5.3) | 129 (3.5) |  |
| Never | 616 (15.2) | 114 (33.8) | 502 (13.5) |  |
| Frequency of taking part in some social activities at present |  |  |  |  |
| Almost everyday | 169 (4.2) | 11 (3.3) | 158 (4.3) | 8 (0.2) |
| Not daily, but once for a week | 112 (2.8) | 3 (0.9) | 109 (2.9) |  |
| Not weekly, but at least once for a month | 100 (2.5) | 7 (2.1) | 93 (2.5) |  |
| Not monthly, but sometimes | 417 (10.3) | 28 (8.3) | 389 (10.5) |  |
| Never | 3241 (80.2) | 287 (85.4) | 2954 (79.8) |  |

**Table S5.** Cohort characteristics – dietary factors

|  | No. (%) | | | |
| --- | --- | --- | --- | --- |
| **Characteristic** |  | **Developed Cognitive Impairments?** | | **Missing** |
|  | Everyone (n = 4047) | Yes (n = 337) | No (n = 3710) |  |
| Staple food |  |  |  |  |
| Rice | 2462 (60.9) | 200 (59.3) | 2262 (61) | 1 (0.0) |
| Corn(maize) | 142 (3.5) | 12 (3.6) | 130 (3.5) |  |
| Wheat (noodles and bread etc.) | 944 (23.3) | 86 (25.5) | 858 (23.1) |  |
| Rice and wheat | 487 (12) | 39 (11.6) | 448 (12.1) |  |
| Other | 11 (0.3) | 0 (0) | 11 (0.3) |  |
| Frequency of eating fresh fruits |  |  |  |  |
| Almost everyday | 567 (14) | 42 (12.5) | 525 (14.2) | 3 (0.1) |
| Quite often | 1040 (25.7) | 81 (24) | 959 (25.9) |  |
| Occasionally | 1466 (36.3) | 132 (39.2) | 1334 (36) |  |
| Rarely or never | 971 (24) | 82 (24.3) | 889 (24) |  |
| Frequency of eating vegetables |  |  |  |  |
| Almost everyday | 2477 (61.4) | 183 (54.3) | 2294 (62) | 11 (0.3) |
| Except winter | 1247 (30.9) | 117 (34.7) | 1130 (30.5) |  |
| Occasionally | 243 (6) | 27 (8) | 216 (5.8) |  |
| Rarely or never | 69 (1.7) | 10 (3) | 59 (1.6) |  |
| Main flavor has |  |  |  |  |
| Insipidity | 2559 (63.4) | 204 (60.9) | 2355 (63.7) | 13 (0.3) |
| Salty | 817 (20.3) | 57 (17) | 760 (20.5) |  |
| Sweet | 191 (4.7) | 25 (7.5) | 166 (4.5) |  |
| Hot | 147 (3.6) | 10 (3) | 137 (3.7) |  |
| Crude | 8 (0.2) | 0 (0) | 8 (0.2) |  |
| Do not have all the above tastes | 312 (7.7) | 39 (11.6) | 273 (7.4) |  |
| Drink or not at present |  |  |  |  |
| Yes | 812 (20.2) | 54 (16.2) | 758 (20.6) | 37 (0.9) |
| No | 3198 (79.8) | 280 (83.8) | 2918 (79.4) |  |
| Type of alcohol consumed |  |  |  |  |
| Never Drink alcohol | 3198 (79.8) | 280 (83.8) | 2918 (79.4) | 39 (1.0) |
| Very strong liquor | 345 (8.6) | 20 (6) | 325 (8.8) |  |
| Not very strong liquor | 197 (4.9) | 21 (6.3) | 176 (4.8) |  |
| Wine | 18 (0.4) | 1 (0.3) | 17 (0.5) |  |
| Rice wine | 140 (3.5) | 8 (2.4) | 132 (3.6) |  |
| Beer | 78 (1.9) | 3 (0.9) | 75 (2) |  |
| Others | 32 (0.8) | 1 (0.3) | 31 (0.8) |  |
| Alcohol consumption, mean (SD), Liang | 0.7 (2) | 0.3 (0.9) | 0.7 (2) | 51 (1.3) |

|  | No. (%) | | | |
| --- | --- | --- | --- | --- |
| **Characteristic** |  | **Developed Cognitive Impairments?** | | **Missing** |
|  | Everyone (n = 4047) | Yes (n = 337) | No (n = 3710) |  |
| Frequency of eating meat at present |  |  |  |  |
| Almost everyday | 1234 (30.5) | 103 (30.7) | 1131 (30.5) | 4 (0.1) |
| Not everyday, but at least once per week | 1786 (44.2) | 149 (44.3) | 1637 (44.2) |  |
| Not every week, but at least once per month | 477 (11.8) | 39 (11.6) | 438 (11.8) |  |
| Not every month, but occasionally | 265 (6.6) | 28 (8.3) | 237 (6.4) |  |
| Rarely or never | 281 (7) | 17 (5.1) | 264 (7.1) |  |
| Frequency of eating fish at present |  |  |  |  |
| Almost everyday | 324 (8) | 14 (4.2) | 310 (8.4) | 2 (0.0) |
| Not every day, but at least once per week | 1536 (38) | 113 (33.6) | 1423 (38.4) |  |
| Not every week, but at least once per month | 891 (22) | 85 (25.3) | 806 (21.7) |  |
| Not every month, but occasionally | 568 (14) | 55 (16.4) | 513 (13.8) |  |
| Rarely or never | 726 (17.9) | 69 (20.5) | 657 (17.7) |  |
| Frequency of eating eggs at present |  |  |  |  |
| Almost everyday | 1243 (30.7) | 98 (29.2) | 1145 (30.9) | 4 (0.1) |
| Not every day, but at least once per week | 1589 (39.3) | 125 (37.2) | 1464 (39.5) |  |
| Not every week, but at least once per month | 466 (11.5) | 41 (12.2) | 425 (11.5) |  |
| Not every month, but occasionally | 322 (8) | 28 (8.3) | 294 (7.9) |  |
| Rarely or never | 423 (10.5) | 44 (13.1) | 379 (10.2) |  |
| Frequency of eating sugar at present |  |  |  |  |
| Almost everyday | 474 (11.7) | 51 (15.2) | 423 (11.4) | 8 (0.2) |
| Not every day, but at least once per week | 793 (19.6) | 67 (19.9) | 726 (19.6) |  |
| Not every week, but at least once per month | 515 (12.8) | 46 (13.7) | 469 (12.7) |  |
| Not every month, but occasionally | 807 (20) | 68 (20.2) | 739 (20) |  |
| Rarely or never | 1450 (35.9) | 104 (31) | 1346 (36.3) |  |

|  | No. (%) | | | |
| --- | --- | --- | --- | --- |
| **Characteristic** |  | **Developed Cognitive Impairments?** | | **Missing** |
|  | Everyone (n = 4047) | Yes (n = 337) | No (n = 3710) |  |
| Frequency of drinking tea at present |  |  |  |  |
| Almost everyday | 1122 (27.8) | 74 (22) | 1048 (28.3) | 7 (0.2) |
| Not everyday, but at least once per week | 276 (6.8) | 20 (6) | 256 (6.9) |  |
| Not every week, but at least once per month | 124 (3.1) | 6 (1.8) | 118 (3.2) |  |
| Not every month, but occasionally | 300 (7.4) | 26 (7.7) | 274 (7.4) |  |
| Rarely or never | 2218 (54.9) | 210 (62.5) | 2008 (54.2) |  |

**Table S6.** Cohort characteristics – psychological factors

|  | No. (%) | | | |
| --- | --- | --- | --- | --- |
| **Characteristic** |  | **Developed Cognitive Impairments?** | | **Missing** |
|  | Everyone (n = 4047) | Yes (n = 337) | No (n = 3710) |  |
| Look on the bright side of things |  |  |  |  |
| Always | 510 (12.7) | 34 (10.2) | 476 (12.9) | 31 (0.8) |
| Often | 2673 (66.6) | 219 (66) | 2454 (66.6) |  |
| Sometimes | 663 (16.5) | 63 (19) | 600 (16.3) |  |
| Seldom | 159 (4) | 16 (4.8) | 143 (3.9) |  |
| Never | 11 (0.3) | 0 (0) | 11 (0.3) |  |
| Keep my belongings neat and clean |  |  |  |  |
| Always | 475 (11.8) | 39 (11.6) | 436 (11.8) | 14 (0.3) |
| Often | 2405 (59.6) | 188 (56) | 2217 (60) |  |
| Sometimes | 1083 (26.9) | 98 (29.2) | 985 (26.6) |  |
| Seldom | 66 (1.6) | 10 (3) | 56 (1.5) |  |
| Never | 4 (0.1) | 1 (0.3) | 3 (0.1) |  |
| Feel fearful or anxious |  |  |  |  |
| Always | 16 (0.4) | 1 (0.3) | 15 (0.4) | 34 (0.8) |
| Often | 118 (2.9) | 8 (2.4) | 110 (3) |  |
| Sometimes | 688 (17.1) | 65 (19.5) | 623 (16.9) |  |
| Seldom | 1639 (40.8) | 139 (41.6) | 1500 (40.8) |  |
| Never | 1552 (38.7) | 121 (36.2) | 1431 (38.9) |  |
| Feel lonely and isolated |  |  |  |  |
| Always | 47 (1.2) | 5 (1.5) | 42 (1.1) | 33 (0.8) |
| Often | 168 (4.2) | 8 (2.4) | 160 (4.3) |  |
| Sometimes | 743 (18.5) | 79 (23.7) | 664 (18) |  |
| Seldom | 1457 (36.3) | 126 (37.7) | 1331 (36.2) |  |
| Never | 1599 (39.8) | 116 (34.7) | 1483 (40.3) |  |
| Make own decision |  |  |  |  |
| Always | 1759 (44.1) | 108 (32.9) | 1651 (45.1) | 61 (1.5) |
| Often | 1079 (27.1) | 82 (25) | 997 (27.3) |  |
| Sometimes | 711 (17.8) | 75 (22.9) | 636 (17.4) |  |
| Seldom | 301 (7.6) | 45 (13.7) | 256 (7) |  |
| Never | 136 (3.4) | 18 (5.5) | 118 (3.2) |  |
| Feel useless with age |  |  |  |  |
| Always | 246 (6.2) | 22 (6.7) | 224 (6.1) | 56 (1.4) |
| Often | 570 (14.3) | 56 (17.1) | 514 (14) |  |
| Sometimes | 1368 (34.3) | 115 (35.1) | 1253 (34.2) |  |
| Seldom | 1035 (25.9) | 79 (24.1) | 956 (26.1) |  |
| Never | 772 (19.3) | 56 (17.1) | 716 (19.5) |  |

|  | No. (%) | | | |
| --- | --- | --- | --- | --- |
| **Characteristic** |  | **Developed Cognitive Impairments?** | | **Missing** |
|  | Everyone (n = 4047) | Yes (n = 337) | No (n = 3710) |  |
| Happier when younger |  |  |  |  |
| Always | 1614 (41.3) | 123 (38.7) | 1491 (41.5) | 140 (3.5) |
| Often | 491 (12.6) | 39 (12.3) | 452 (12.6) |  |
| Sometimes | 913 (23.4) | 73 (23) | 840 (23.4) |  |
| Seldom | 702 (18) | 65 (20.4) | 637 (17.7) |  |
| Never | 187 (4.8) | 18 (5.7) | 169 (4.7) |  |
| Whether felt sad, blue, or depressed for two weeks or more in last 12 months |  |  |  |  |
| Yes | 522 (13.8) | 39 (12.9) | 483 (13.9) | 266 (6.6) |
| No | 3259 (86.2) | 264 (87.1) | 2995 (86.1) |  |

**Table S7.** Cohort characteristics – exercise and sleep factors

|  | No. (%) | | | |
| --- | --- | --- | --- | --- |
| **Characteristic** |  | **Developed Cognitive Impairments?** | | **Missing** |
|  | Everyone (n = 4047) | Yes (n = 337) | No (n = 3710) |  |
| Exercise or not at present |  |  |  |  |
| Yes | 1670 (41.8) | 114 (34.3) | 1556 (42.4) | 47 (1.2) |
| No | 2330 (58.2) | 218 (65.7) | 2112 (57.6) |  |
| Exercised or not in the past |  |  |  |  |
| Yes | 1066 (26.6) | 86 (25.7) | 980 (26.7) | 37 (0.9) |
| No | 2944 (73.4) | 248 (74.3) | 2696 (73.3) |  |
| Quality of sleep |  |  |  |  |
| Very good | 775 (19.2) | 60 (17.8) | 715 (19.3) | 2 (0.0) |
| Good | 1804 (44.6) | 169 (50.1) | 1635 (44.1) |  |
| So so | 973 (24.1) | 77 (22.8) | 896 (24.2) |  |
| Bad | 450 (11.1) | 31 (9.2) | 419 (11.3) |  |
| Very bad | 43 (1.1) | 0 (0) | 43 (1.2) |  |
| Duration of sleeping, mean (SD), hour | 7.5 (2.1) | 8 (2.3) | 7.4 (2) | 12 (0.3) |

**Table S8.** Cohort characteristics – activities of daily living factors

|  | No. (%) | | | |
| --- | --- | --- | --- | --- |
| **Characteristic** |  | **Developed Cognitive Impairments?** | | **Missing** |
|  | Everyone (n = 4047) | Yes (n = 337) | No (n = 3710) |  |
| Bathing |  |  |  |  |
| Without assistance | 3768 (93.2) | 280 (83.1) | 3488 (94.2) | 6 (0.1) |
| One part assistance | 134 (3.3) | 23 (6.8) | 111 (3) |  |
| More than one part assistance | 139 (3.4) | 34 (10.1) | 105 (2.8) |  |
| Dressing |  |  |  |  |
| Without assistance | 3967 (98.5) | 313 (93.7) | 3654 (99) | 21 (0.5) |
| Need assistance for trying shoes | 18 (0.4) | 8 (2.4) | 10 (0.3) |  |
| Assistance in getting clothes and getting dressed | 41 (1) | 13 (3.9) | 28 (0.8) |  |
| Toileting |  |  |  |  |
| Without assistance | 3971 (98.4) | 312 (92.9) | 3659 (98.9) | 10 (0.2) |
| Assistance in cleaning or arranging clothes | 53 (1.3) | 18 (5.4) | 35 (0.9) |  |
| Don't use toilet | 13 (0.3) | 6 (1.8) | 7 (0.2) |  |
| Indoor transferring |  |  |  |  |
| Without assistance | 3970 (98.7) | 315 (94.3) | 3655 (99.1) | 26 (0.6) |
| With assistance | 45 (1.1) | 16 (4.8) | 29 (0.8) |  |
| Bedridden | 6 (0.1) | 3 (0.9) | 3 (0.1) |  |
| Continence |  |  |  |  |
| Without assistance | 3985 (98.7) | 326 (96.7) | 3659 (98.9) | 10 (0.2) |
| Occasional accidents | 46 (1.1) | 9 (2.7) | 37 (1) |  |
| Incontinent | 6 (0.1) | 2 (0.6) | 4 (0.1) |  |
| Feeding |  |  |  |  |
| Without assistance | 3968 (99.3) | 324 (96.1) | 3644 (99.6) | 50 (1.2) |
| With some help | 27 (0.7) | 12 (3.6) | 15 (0.4) |  |
| Need feeding | 2 (0.1) | 1 (0.3) | 1 (0) |  |

**Table S9.** Cohort characteristics – chronic disease risk factors

|  | No. (%) | | | |
| --- | --- | --- | --- | --- |
| **Characteristic** |  | **Developed Cognitive Impairments?** | | **Missing** |
|  | Everyone (n = 4047) | Yes (n = 337) | No (n = 3710) |  |
| Suffering from hypertension |  |  |  |  |
| Yes | 1289 (32.8) | 108 (33) | 1181 (32.8) | 120(3.0) |
| No | 2638 (67.2) | 219 (67) | 2419 (67.2) |  |
| Suffering from diabetes |  |  |  |  |
| Yes | 192 (4.9) | 9 (2.8) | 183 (5.1) | 168(4.2) |
| No | 3687 (95.1) | 307 (97.2) | 3380 (94.9) |  |
| Suffering from heart disease |  |  |  |  |
| Yes | 520 (13.4) | 46 (14.3) | 474 (13.3) | 153 (3.8) |
| No | 3374 (86.6) | 275 (85.7) | 3099 (86.7) |  |
| Suffering from stroke or cvd |  |  |  |  |
| Yes | 275 (7) | 17 (5.3) | 258 (7.2) | 139 (3.4) |
| No | 3633 (93) | 306 (94.7) | 3327 (92.8) |  |
| Suffering from blood disease |  |  |  |  |
| Yes | 167 (4.5) | 7 (2.3) | 160 (4.7) | 344 (8.5) |
| No | 3536 (95.5) | 297 (97.7) | 3239 (95.3) |  |
